# TET2 functions as a resistance factor against DNA methylation acquisition during Epstein-Barr virus infection

**DOI:** 10.18632/oncotarget.13130

**Published:** 2016-11-05

**Authors:** Hiroe Namba-Fukuyo, Sayaka Funata, Keisuke Matsusaka, Masaki Fukuyo, Bahityar Rahmutulla, Yasunobu Mano, Masashi Fukayama, Hiroyuki Aburatani, Atsushi Kaneda

**Affiliations:** ^1^ Department of Molecular Oncology, Graduate School of Medicine, Chiba University, Japan; ^2^ Department of Computational Biology and Medical Sciences, Graduate School of Frontier Sciences, The University of Tokyo, Japan; ^3^ Japan Society for the Promotion of Science, Japan; ^4^ Department of Pathology, Graduate School of Medicine, The University of Tokyo, Japan; ^5^ Genome Science Division, Research Center for Advanced Science and Technology, The University of Tokyo, Japan

**Keywords:** DNA hydroxymethylation, DNA methylation, Epstein-Barr virus, gastric cancer, TET2

## Abstract

Extensive DNA methylation is observed in gastric cancer with Epstein-Barr virus (EBV) infection, and EBV infection is the cause to induce this extensive hypermethylaton phenotype in gastric epithelial cells. However, some 5′ regions of genes do not undergo *de novo* methylation, despite the induction of methylation in surrounding regions, suggesting the existence of a resistance factor against DNA methylation acquisition. We conducted an RNA-seq analysis of gastric epithelial cells with and without EBV infection and found that TET family genes, especially *TET2*, were repressed by EBV infection at both mRNA and protein levels. *TET2* was found to be downregulated by EBV transcripts, e.g. BARF0 and *LMP2A*, and also by seven human miRNAs targeting *TET2*, e.g., miR-93 and miR-29a, which were upregulated by EBV infection, and transfection of which into gastric cells repressed *TET2*. Hydroxymethylation target genes by TET2 were detected by hydroxymethylated DNA immunoprecipitation sequencing (hMeDIP-seq) with and without TET2 overexpression, and overlapped significantly with methylation target genes in EBV-infected cells. When *TET2* was knocked down by shRNA, EBV infection induced *de novo* methylation more severely, including even higher methylation in methylation-acquired promoters or *de novo* methylation acquisition in methylation-protected promoters, leading to gene repression. *TET2* knockdown alone without EBV infection did not induce *de novo* DNA methylation. These data suggested that TET2 functions as a resistance factor against DNA methylation in gastric epithelial cells and repression of *TET2* contributes to DNA methylation acquisition during EBV infection.

## INTRODUCTION

Aberrant DNA methylation is one of the major epigenomic alterations, and DNA hypermethylation of gene promoter regions inactivates tumor suppressor genes and strongly affects cancer development [1–3]. Epstein-Barr virus (EBV)-positive gastric cancer shows a specific hypermethylation phenotype [4–8], which is reportedly the most extensive hypermethylation phenotype among all human malignancies [9]. By infecting low-methylation gastric cancer cells with EBV *in vitro*, previous studies have demonstrated that EBV infection itself causes the induction of extensive hypermethylation [7, 10, 11].

The molecular mechanism of methylation induction during EBV infection is largely unknown. Latent EBV infection upregulates DNA methyltransferases (DNMTs), resulting in extensive methylation in the EBV genome and, subsequently, in the host genome. The EBV protein, latent membrane protein 2A (LMP2A) induces DNMT1 by phosphorylating STAT3 [12]. EBV infection downregulates the expression of the host miR-200 family, which targets *ZEB1* and *ZEB2*; the upregulation of ZEB1 and ZEB2 results in *CDH1* repression [13].

Other than gastric cancer, leukemia and glioma also possess high methylation epigenotype. Mutations in the TET (ten-eleven-translocation) family gene *TET2* have been observed in 15% of various myeloid cancer patients; these mutations lead to DNA hypermethylation and induce leukemogenesis [14, 15]. TET family genes encode DNA demethylases that oxidize 5-methylcytosine (5mC) to 5-hydroxymethylcytosine (5hmC), 5-formylcytosine (5fC), and finally 5-carboxylcytosine (5caC) [16, 17]. By base excision repair via thymine DNA glycosylase, 5fC and 5caC are directly changed to unmodified cytosine [18]. The subgroup of glioma with extensive promoter hypermethylation is known as glioma CpG island methylator phenotype (G-CIMP) [19]. More than 70% of low-grade gliomas (grades II and III) possess mutations in *IDH1* or *IDH2*. The mutant proteins produce D-2-hydroxyglutarate and inhibit α-ketoglutarate, a cofactor of TET family proteins, and thus inhibit the TET-induced hydroxymethylation of DNA [20–22].

To investigate the molecular mechanism by which extensive hypermethylation is induced in EBV-positive gastric cancer, we conducted a transcriptome analysis, and *TET2* was found to be one of the downregulated genes. Hydroxymethylation target genes induced by TET2 were significantly overlapped with methylation target genes during EBV infection. When *TET2* was knocked down, significantly more genes acquired promoter hypermethylation and were repressed. We here show an important role of TET2 as a resistance factor against *de novo* methylation during EBV infection and the contribution of TET2 downregulation to DNA methylation acquisition.

## RESULTS

### Transcriptome analysis

**Figure 1:**
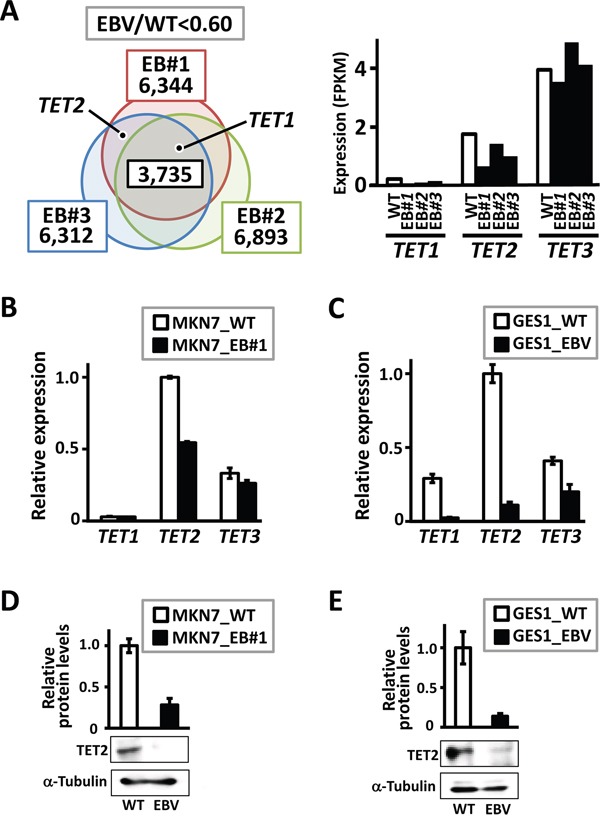
Downregulation of genes in the TET family in EBV-infected cells **A.** Genome-wide gene expression levels were analyzed by RNA-seq. The diagram indicates the numbers of genes downregulated by <0.6-fold in three EBV-infected clones compared with MKN7_WT, i.e., 6,344 genes in MKN7_EB#1, 6,893 genes in MKN7_EB#2, and 6,312 genes in MKN7_EB#3. *TET1* was included in the 3,735 genes that were downregulated in all the three clones, and *TET2* was downregulated in MKN7_EB#1 and EB#3. **B.** Expression levels of *TET* genes were validated by real-time RT-PCR, and normalized against that of *GAPDH*. *TET2* expression was markedly decreased in MKN7_EB#1, while *TET1* expression was very low in both MKN7_WT and MKN7_EB#1. The experiment was done twice to confirm the similar result. **C.** Expression levels of *TET* genes were also analyzed in GES1, and normalized against that of *GAPDH*. All *TET* genes, especially *TET2*, showed marked decreases in GES1_EBV compared with GES1_WT. The experiment was done twice to confirm the similar result. **D, E.** Immunoblotting analysis was conducted for TET2 and α-Tubulin in MKN7_WT and MKN7_EB#1 (*D*) and GES1_WT and GES1_EBV (*E*), and the analysis was done twice to confirm the similar result. The ratio of the intensity of TET2, measured by densitometer, to that of α-Tubulin was shown as a relative expression level.

To identify candidate resistance factors for methylation acquisition during EBV infection, we performed an RNA-seq analysis using a low-methylation gastric cancer cell line, MKN7 (MKN7_WT), and three previously established EBV-infected MKN7 clones (MKN7_EB#1, EB#2, and EB#3) [7]. Downregulated genes in response to EBV infection included *TET1* and *TET2*, which encode TET family demethylation enzymes (Figure 1A). Quantitative RT-PCR to analyze TET family genes was performed using MKN7_WT and MKN7_EB#1. *TET2* was markedly downregulated after EBV infection, and *TET1* was expressed at low levels in both cells (Figure 1B). Another gastric epithelial cell line, GES1 (GES1_WT), established from normal gastric epithelial cells, was also infected with EBV (GES1_EBV). This cell line acquires extensive hypermethylation in response to *in vitro* EBV infection (Matsusaka et al., *unpublished data*). Based on a quantitative RT-PCR analysis, the three *TET* family genes were downregulated in GES1_EBV, especially *TET2* (Figure 1C). Immunoblotting analyses also showed that TET2 protein expression was significantly repressed by EBV infection in both MKN7 (Figure 1D) and GES1 cells (Figure 1E). Since *TET2* expression was markedly decreased after EBV infection in both MKN7 and GES1 cells among the three *TET* family genes, and TET2 is involved in cytosine hydroxymethylation, we hypothesized that *TET2* downregulation contributes to methylation, at least partially.

As for other epigenetic modifiers, downregulation of *HDAC8* and upregulation of *SUZ12* and *BMI1* were observed in EBV-infected clones (Supplementary Figure S1).

### Downregulation of TET2

**Figure 2:**
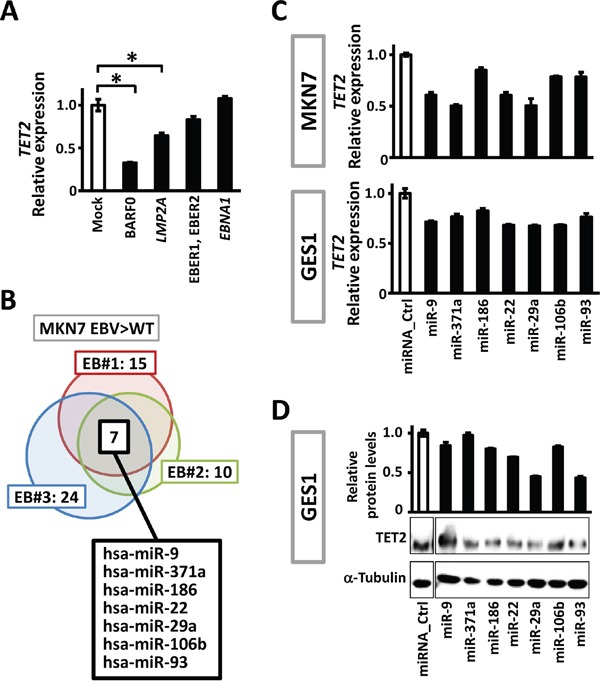
Viral and cellular factors to downregulate TET2 **A.** Expression level of *TET2* was analyzed in MKN7 overexpressing EBV latent genes, *LMP2A*, *ENBA1*, BARF0 and EBER1/2, and normalized against that of *GAPDH*. BARF0 and *LMP2A* significantly downregulated *TET2*. **B.** Expression of 2,549 human miRNAs in MKN7_WT and the three EBV-infected clones were analyzed using a microarray. Among 87 miRNAs that were expected to target *TET2* based on an *in silico* analysis, 15, 10, and 24 miRNAs were upregulated >1.3-fold in MKN7_EB#1, EB#2, and EB#3, respectively, and 7 miRNAs were upregulated in all the three clones. **C.** The 7 miRNAs were transfected into MKN7 and GES1, and real-time RT-PCR showed that *TET2* expression levels decreased by 50-85% after 48 h. The experiment was done twice to confirm the similar result. **D.** Immunoblotting analysis was conducted for TET2 and α-Tubulin expression in GES1 transfected with the 7 miRNAs.

To investigate the mechanism which downregulates *TET2* expression during EBV infection, we first examined if EBV encoded transcripts contribute to decrease of *TET2*. It is known that most of EBV genome is dense methylated in latent infection in gastric epithelial cells, and the limited number of protein-coding genes, *LMP2A* and *EBNA1*, and non-coding transcripts, BARF0 and EBER1/2, are allowed to express [5, 23]. These are called latent genes, and were overexpressed in MKN7 cells. It was found that *TET2* was downregulated to 0.35-fold by BARF0, and 0.65-fold by *LMP2A* (Figure 2A).

To examine effects of cellular transcripts on *TET2* expression, we next conducted miRNA microarray analysis for human miRNA expression in MKN7_WT and three EBV-infected MKN7 clones. Of 83 candidate miRNAs that targeted *TET2* according to *in silico* data (http://microrna.org/), 7 miRNAs were commonly upregulated in the three EBV-infected MKN7 clones compared to MKN7_WT (Figure 2B). To validate whether these 7 miRNAs decrease *TET2* expression, we transfected the miRNAs into MKN7 and another cell line GES1 and performed quantitative RT-PCR to analyze *TET2*. All the 7 miRNAs decreased *TET2* expression levels to 50–85% in MKN7 as well as GES1 cells, suggesting that the upregulation of these 7 miRNAs downregulates *TET2*, at least partly (Figure 2C). Downregulation of TET2 protein was also confirmed by immunoblotting analysis (Figure 2D). Transfection of miR-29a and miR93 induced more marked downregulation in protein level than in mRNA level, which is consistent with reports explaining that miRNA works for not only mRNA cleavage but also translational repression [24]. The predicted regions for the 7 miRNAs to bind 3′ UTR of *TET2* were analyzed using microRNA.org [25] and shown (Supplementary Figure S2).

We next searched for human genes targeted by viral miRNAs encoded in EBV genome, using computational software Vir-Mir database [26, 27]. No EBV miRNA was found to target human *TET2* (Supplementary Table S1).

### Hydroxymethylation target genes of TET2

**Figure 3:**
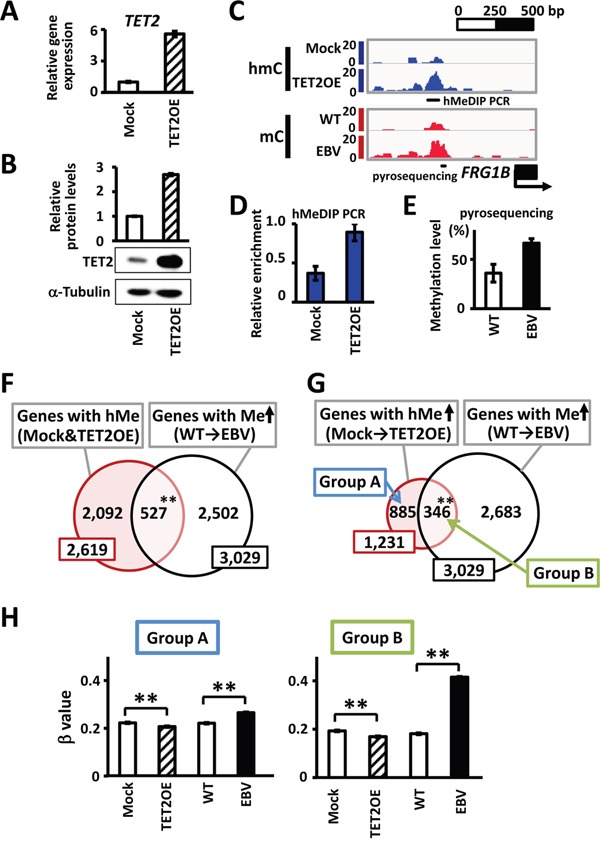
Hydroxymethylation target genes by TET2 **A.** A *TET2*-expressing vector was transfected into GES1 and the expression level of *TET2* relative to *GAPDH* at 30 days after transfection was analyzed by real-time RT-PCR. *Mock*, GES1 cells transfected with an empty vector as negative controls. *TET2OE*, GES1 overexpressing *TET2*. **B.** Immunoblotting analysis was conducted for TET2 and α-Tubulin expression in Mock and TET2OE cells. **C.** Representative results of hMeDIP-seq and MeDIP-seq around *FRG1B* are shown. The hydroxymethylation level of the region was increased in cells with TET2 overexpression, whereas the methylation level was increased in EBV infection. **D.** hMeDIP was repeated, and increase of hmC in 5′ region of *FRG1B* was validated by hMeDIP-PCR at the region indicated in Figure 3C, and normalized against a positive control region *NEDD9*. **E.** Increase of mC was validated by quantitative pyrosequencing assay at the region indicated in Figure 3C. **F.** Among 2,619 hydroxymethylation target genes showing hydroxymethylation peaks within ±1 kb of the TSS in both Mock and TET2OE cells, 527 genes (20.1%) were methylation target genes during EBV infection (*P*<1×10^-15^). **G.** Among hydroxymethylation target genes in TET2OE, 1,231 genes showing increased hydroxymethylation from Mock to TET2OE were extracted as hydroxymethylation target genes by TET2. As many as 346 genes (28.1%) were methylation target genes during EBV infection (*P*<1×10^-15^). **H.** Methylation levels of hydroxymethylation target genes by TET2 were analyzed by Infinium, and average β values are shown. The 346 methylation target genes during EBV infection (*Group B*) showed marked increases of β values in GES1_EBV, while the other 885 genes that were not extracted as methylation target genes during EBV infection (*Group A*) showed slight, but still significant increases of β values in GES1_EBV. Both genes in Group A and Group B showed decreases of β values in TET2OE. *WT*, GES1_WT. *EBV*, GES1_EBV.

To identify hydroxymethylation target genes in response to TET2, we performed hMeDIP-seq and MeDIP-seq analyses using GES1 cells transfected with a *TET2*-overexpression vector (TET2OE) or mock vector (Mock) (Figure 3A-3E). We detected 2,619 hydroxymethylation target genes that showed 5hmC peaks in the hMeDIP-seq analysis in both Mock and TET2OE cells. Among 2,619 genes, a significant number of genes overlapped with the 3,029 methylation target genes in EBV-infected cells (527 genes, *P*<1×10^-15^, χ^2^ test) (Figure 3F). These results suggested that many genes remain unmethylated owing to hydroxymethylation by TET2 before EBV infection, leading to methylation via TET2 depression after EBV infection. However, other enzymes, in addition to TET2, may produce 5hmC. To specifically analyze TET2 target genes, we focused on 1,231 genes that did not possess 5hmC peaks in Mock cells, but acquired 5hmC peaks after TET2 overexpression. Among these 1,231 genes, more significant overlap with methylation target genes was found (346 genes, *P*<1×10^-15^, χ^2^ test) (Figure 3G). These 346 methylation target genes during EBV infection (Group B in Figure 3G) showed significant decreases in β values when TET2 was overexpressed (*P*<1×10^-15^), and significant increases after EBV infection (*P*<1×10^-15^) (Figure 3H). The other 885 genes that were not extracted as methylation target genes during EBV infection (Group A in Figure 3G) showed slight, but significant increases in β values in GES1_EBV (*P*<1×10^-15^) (Figure 3H). These results suggested that hydroxymethylation by TET2 protects the unmethylated status of genes before EBV infection, and decreased TET2 via EBV infection could promote the methylation of these target genes.

### Knockdown of TET2 accelerated de novo methylation during EBV infection

**Figure 4:**
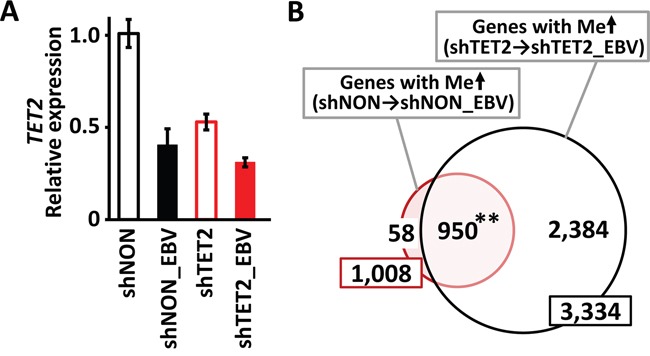
Knockdown of TET2 by shRNA **A.** *TET2* was knocked down in MKN7 by infection with a shRNA lentivirus targeting *TET2* (shTET2). *shNON*, MKN7 cells infected with non-targeting shRNA. Real-time RT-PCR analysis of *TET2*, normalized against *PPIA*, showed that the expression level of *TET2* was lower in shTET2 than shNON cells. After EBV infection of shNON and shTET2 cells (shNON_EBV and shTET2_EBV, respectively), *TET2* levels were lower in shNON_EBV than shNON, and decreased further in shTET2_EBV compared with shTET2. **B.** While 1,008 genes acquired promoter methylation by EBV infection in shNON cells, 3,334 genes showed *de novo* methylation by EBV infection in shTET2 cells, and 950 genes (94.2%) overlapped in the two cell types. “*De novo* methylation” was defined as >2 probes with β <0.2 that increased to β >0.4 within ±1 kb of TSS after EBV infection.

If TET2 is a resistance factor for methylation acquisition, the knockdown of *TET2* might accelerate methylation acquisition during EBV infection. We therefore knocked down *TET2* in MKN7 cells by shRNA (shTET2) and infected shTET2 cells with EBV (shTET2_EBV) (Figure 4A). Non-targeting shRNA lentivirus was also transfected to obtain control cells (shNON). Seventy-two days after EBV infection, DNA methylation levels were quantitatively investigated using the Infinium 450k beadarray. When shNON cells were infected with EBV (shNON_EBV), 1,008 genes acquired *de novo* promoter methylation, whereas 3,334 genes acquired *de novo* promoter methylation when shTET2 cells were infected with EBV (shTET2_EBV), including as many as 950 of the 1,008 genes in shNON-EBV. While most (94%) of the methylation target genes in shNON cells were also methylated in shTET2 cells, 2,384 genes were newly methylated in *TET2*-depleted cells (Figure 4B).

**Figure 5:**
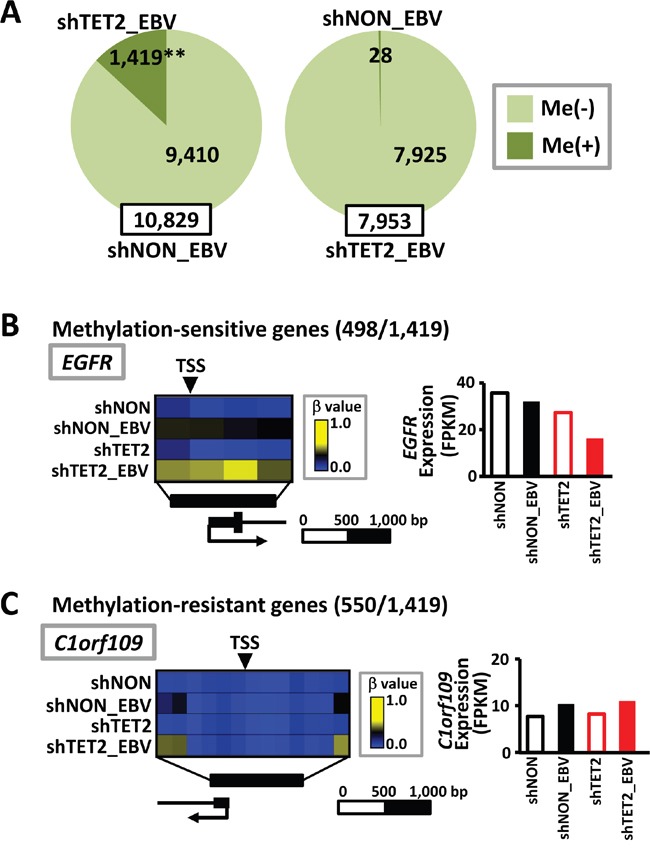
Increase in methylation target genes by EBV infection in TET2-depleted cells **A.** Among 10,829 genes defined as unmethylated in shNON_EBV cells, 1,419 genes acquired methylation in shTET2_EBV. In contrast, only 28 genes showed differential methylation in shNON_EBV among 7,953 genes that were unmethylated in shTET2_EBV (*P*<1×10^-15^). **B.** Among the 1,419 genes showing differential methylation in shTET2_EBV (*A*), 498 genes became methylation-sensitive genes, acquiring complete methylation in the promoter regions. Representative methylation levels in the *EGFR* promoter region are shown. **C.** Among the 1,419 genes showing differential methylation in shTET2_EBV (*A*), 550 were methylation-resistant genes, acquiring methylation in the regions surrounding promoters, but maintaining an unmethylated status in narrow regions around the TSS. The methylation levels of the *C1orf109* promoter region are representatively shown.

Next, among 10,829 genes that were defined as unmethylated in shNON_EBV cells, 1,419 genes acquird *de novo* methylation in shTET2_EBV cells. In contrast, among 7,953 genes defined as unmethylated in shTET2_EBV cells, only 28 showed *de novo* methylation in shNON_EBV (*P*<1×10^-15^) (Figure 5A). Among the 1,419 genes that showed *de novo* methylation in shTET2_EBV cells, 498 were methylation-sensitive genes that acquired complete methylation in their promoter regions (Figure 5B), and 550 were methylation-resistant genes that underwent *de novo* methylation in the region surrounding the TSS, but maintained an unmethylated status in narrow regions around the TSS (Figure 5C, Supplementary Figure S3). The 498 methylation-sensitive genes, e.g., *EGFR,* showed decreased expression in shTET2_EBV cells, but the 550 methylation-resistant genes, e.g., *C1orf109,* did not show decreased expression in shTET2_EBV cells (Figure 5B and 5C).

**Figure 6:**
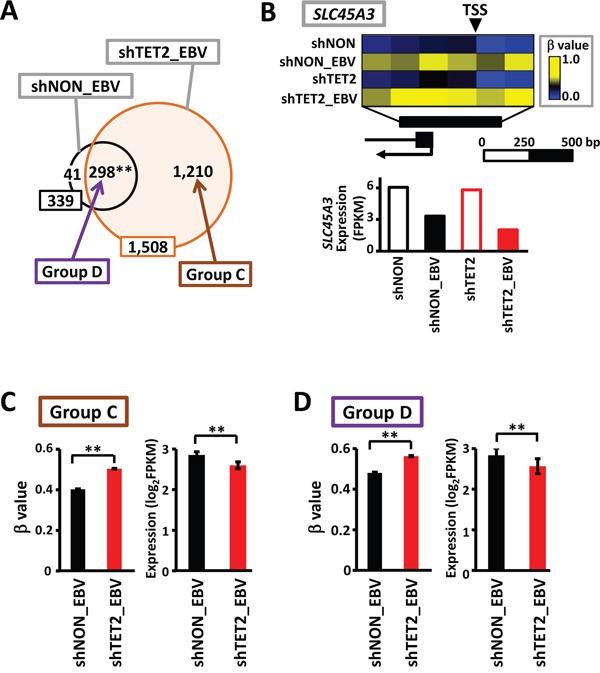
Increase in methylation levels, even in methylation-sensitive genes, by TET2 knockdown **A.** The number of methylation-sensitive genes was 339 in shNON_EBV, and this increased to 1,508 in shTET2_EBV, with 298 overlapping genes (87.9%). **B.** *SLC45A3* was one of the 298 genes showing complete promoter methylation in both shNON_EBV and shTET2_EBV. The methylation level was even higher and the expression level was even lower in shTET2_EBV compared with shNON_EBV. **C.** The 1,210 methylation-sensitive genes in shTET2_EBV only (*Group C*) showed significant increases in methylation levels (*P*<1×10^-15^) and significant decreases in expression levels (*P*=2×10^-12^) in comparison with the levels observed in shNON_EBV. **D.** The 298 methylation-sensitive genes in both shNON_EBV and shTET2_EBV (*Group D*) also showed significant increases in methylation levels (*P*<1×10^-15^) and significant decreases in expression levels (*P*=7×10^-5^) in comparison with the levels observed in shNON_EBV.

When methylation-sensitive genes (i.e., genes that were completely methylated in their promoter regions) in shNON_EBV and shTET2_EBV cells were compared, 298 overlapped (Figure 6A and 6B) and 1,210 were methylated only in shTET2_EBV. The 1,210 genes that acquired complete methylation in shTET2_EBV cells only (Group C in Figure 6A), exhibited significantly higher methylation levels (*P*<1×10^-15^, Wilcoxon signed-rank test) and lower expression levels (*P*=2×10^-12^, Wilcoxon signed-rank test) in shTET2_EBV cells than shNON_EBV cells (Figure 6C). Interestingly, the 298 genes that acquired complete methylation in both shNON_EBV and shTET2_EBV cells (Group D in Figure 6A) also showed significantly higher methylation levels (*P*<1×10^-15^, Wilcoxon signed-rank test) and lower expression levels (*P*=7×10^-5^, Wilcoxon signed-rank test) in shTET2_EBV cells than shNON_EBV cells (Figure 6D).

**Figure 7:**
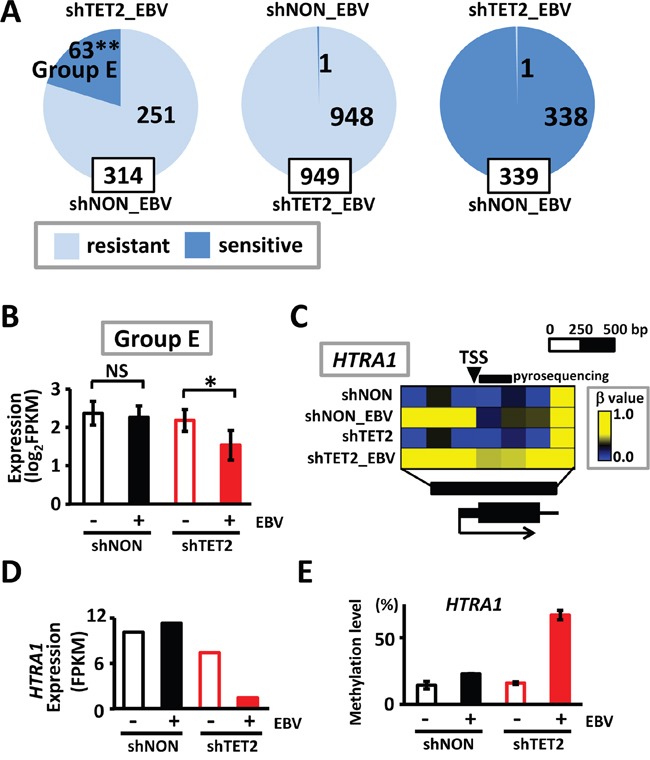
Methylation acquisition in methylation-resistant genes in shNON_EBV **A.** *Resistant*, methylation-resistant. *Sensitive*, methylation-sensitive. Among 314 methylation-resistant genes in shNON_EBV, which showed protection of the unmethylated status around the TSS in shNON_EBV, 63 genes acquired complete promoter methylation in shTET2_EBV, *i.e.*, they became methylation-sensitive by *TET2* depletion (*left*). Among 949 methylation-resistant genes in shTET2_EBV, in contrast, only 1 gene was methylation-sensitive in shNON_EBV (*middle*). Among 339 methylation-sensitive genes in shNON_EBV, only 1 gene became methylation-resistant in shTET2_EBV (*right*). **B.** The 63 genes that became methylation-sensitive in shTET2_EBV (*Group E*) showed significant decreases in expression by EBV infection, only when infected into *TET2*-depleted cells (*P*=0.006). **C.** Representative methylation status of *HTRA1* is shown. *HTRA1* promoter remained unmethylated in shNON_EBV, but acquired complete promoter methylation in shTET2_EBV. **D.** *HTRA1* expression was retained in shNON_EBV, but silenced in shTET2_EBV cells. **E.** Quantitative pyrosequencing assay was conducted to validate unmethylated status in shNON_EBV and acquisition of promoter methylation in shTET2_EBV at the 5′ region of *HTRA1* indicated in Figure 7C.

Among the 314 methylation-resistant genes in shNON_EBV cells that underwent *de novo* methylation in the region surrounding promoters, but maintained an unmethylated status in narrow regions around the TSS, 63 acquired complete methylation in the promoter regions (i.e., were classified as methylation-sensitive genes) in shTET2_EBV (Group E in Figure 7A). These genes did not show decreased expression in shNON_EBV cells, presumably because their TSS was protected from methylation, but they were significantly downregulated in shTET2_EBV cells, presumably because their TSS acquired complete methylation (*P*=0.006) (Figure 7B). Complete acquisition of methylation in *HTRA1* and its repression in shTET2_EBV cells were representatively shown (Figure 7C and 7D), and the methylation changes were validated by pyrosequencing (Figure 7E). Among 949 methylation-resistant genes in shTET2_EBV cells, only 1 gene acquired complete methylation in the promoter region in shNON_EBV cells (*P*<1×10^-15^, χ^2^ test) (Figure 7A). Additionally, only 1 gene became methylation-resistant in shTET2_EBV cells among 339 methylation-sensitive genes in shNON_EBV cells (*P*<1×10^-15^, χ^2^ test) (Figure 7A).

### No methylation was induced by TET2 knockdown alone

**Figure 8:**
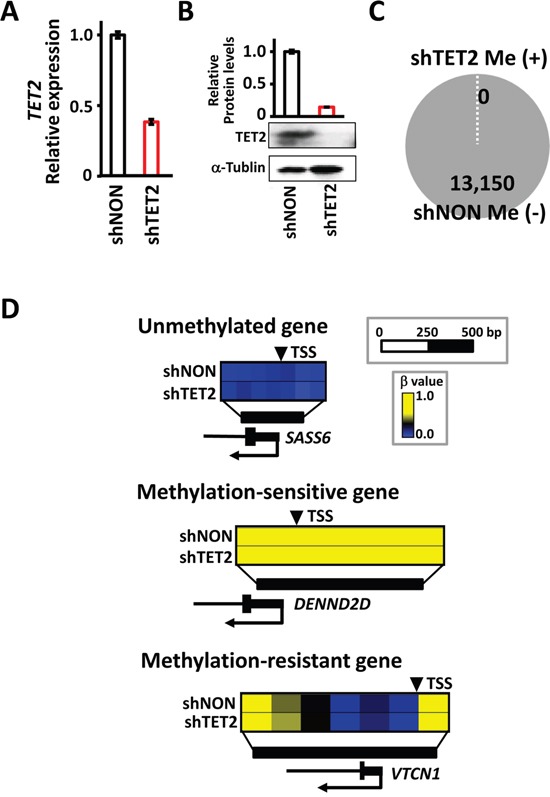
No methylation induction by TET2 knockdown itself, without EBV infection **A.** Real-time RT-PCR showed a decrease in *TET2* expression in shTET2 compared with shNON cells. The experiment was done twice to confirm the similar result. **B.** Immunoblotting analysis was conducted for TET2 and α-Tubulin expression in shNON and shTET2 cells. **C.** Among 13,150 genes that were unmethylated in shNON, none showed methylation in shTET2, *i.e.*, *de novo* methylation did not occur by *TET2* knockdown alone. **D.** Three representative genes showed no methylation alterations in response to the knockdown of *TET2*.

To analyze the effect of *TET2* knockdown, shTET2 and shNON cells were cultured without EBV infection, and methylation alterations were analyzed using the Infinium beadarray (Figure 8). Knockdown of TET2 expression was confirmed in mRNA and protein levels (Figure 8A and 8B). Of 13,150 genes that were defined as unmethylated in shNON cells, none acquired *de novo* methylation in shTET2 cells (Figure 8C). No induction of methylation was detected in unmethylated genes, methylation-sensitive genes, or methylation-resistant genes (Figure 8D). These results suggested that *TET2* depletion is not sufficient to induce *de novo* methylation, and that EBV infection might also trigger other mechanisms in addition to repression of a resistant factor.

## DISCUSSION

We have previously shown that EBV infection causes extensive DNA methylation in gastric epithelial cells [7, 23]. In this study, we performed a transcriptome analysis to identify candidate critical factor(s) that contribute to the epigenetic alterations. *TET2* was downregulated during EBV infection, and this was at least partially caused by expression of EBV transcripts, BARF0 and *LMP2A*, and upregulation of human miRNAs targeting *TET2*.

TET2 is a member of TET family proteins converting DNA 5mC to 5hmC, 5fC, and 5caC [16–18]. *TET2* mutations in various myeloid cancer [14, 15], or mutations of *IDH1* and *IDH2* in low-grade gliomas, which can inhibit DNA hydroxymethylation by TET enzymes [19-22, 28], have been reported and cause DNA hypermethylation phenotype. Here, we showed that *TET2* could be downregulated in gastric epithelial cells via EBV transcripts and upregulation of human miRNAs targeting *TET2*. Other TET family genes are also of great interest since several comprehensive genomic analyses in gastric cancer revealed mutation of *TET1* in microsatellite-stable gastric cancers [8], and *TET1* was also downregulated by EBV infection (Figure 1).

Since a ChIP-seq-grade anti-TET2 antibody is not available, the detection of TET2 binding regions by ChIP-seq was not possible; accordingly, hydroxymethylated DNA regions by TET2 overexpression were detected by hMeDIP-seq. We performed a hMeDIP-seq analysis using Mock and TET2OE cells, and identified hydroxymethylated promoter regions that showed 5hmC peaks in both cells. These hydroxymethylated genes overlapped significantly with methylation target genes in EBV-infected cells. However, hydroxymethylation of these genes is not necessarily caused by TET2, but might be related to other TET family proteins. To detect hydroxymethylated genes converted by TET2, we focused on genes that showed no 5hmC peaks in Mock cells, but were hydroxymethylated in TET2OE cells. These hydroxymethylation target genes by TET2 showed markedly significant overlap with methylation target genes in EBV-infected cells. These results suggested that hydroxymethylation by TET2 is involved in protecting DNA from methylation, and that the repression of this resistance factor against DNA methylation may contribute to *de novo* methylation acquisition during EBV infection.

When *TET2* was knocked down, more genes acquired *de novo* methylation by EBV infection, including genes that were resistant to *de novo* methylation around TSS in shNON_EBV cells (Figure 7). This also indicated that TET2 functions as a resistance factor against DNA methylation during EBV infection, and *TET2* depletion leads to a loss of protective mechanisms and the acquisition of *de novo* methylation. But these genes were not necessarily hydroxymethylation target genes in shNON_EBV cells (data not shown). It is possible that these regions could be further oxidized to 5fC and 5caC, which would be excised by thymine DNA glycosylase to be converted to unmodified cytosine [16-18, 29]. Furthermore, TET1, TET2, and TET3 reportedly bind to DNA without the appearance of 5mC or 5hmC, indicating that they may protect DNA from methylation by physically binding to DNA, regardless of their catalytic activity [30, 31].

Interestingly, *TET2* knockdown itself did not induce methylation. It has been suggested that additional factor(s) are required to induce *de novo* methylation owing, for example, to the increase in methylation pressure via upregulated DNMTs [7], and that TET2 might function in resistance against those factors. EBV infection might also trigger this pressure, and methylation might not be induced without EBV infection, even if the resistant factor is depleted. In transcriptome analysis, histone deacetylase *HDAC8* was downregulated in EBV-infected clones, and genes encoding Polycomb group proteins such as *SUZ12* and *BMI1* were upregulated in EBV-infected clones. Expression changes of these epigenetic factors might also be important for epigenomic alteration after EBV infection. To fully clarify the molecular mechanism underlying the unique epigenomic phenotype with extensive hypermethylation in EBV-positive gastric cancer, further investigations are necessary to identify factors that induce methylation pressure and to determine how the factors are activated and recruited to methylation target genes.

In summary, we found that *TET2* is downregulated during EBV infection via expression of EBV transcripts and upregulation of human miRNAs targeting *TET2*, and that TET2 may function as a resistance factor against DNA methylation in gastric epithelial cells. While TET2 depletion itself does not increase the methylation level, the downregulation of *TET2* contributes to methylation induction by EBV infection.

## MATERIALS AND METHODS

### Cell culture and treatment

The gastric cancer cell line MKN7 was obtained from Riken BioResource Center Cell Bank and was authenticated by the cell bank using short tandem repeat PCR. The normal fetal gastric mucosal cell line GES1, which was immortalized by SV40, was obtained from the Beijing Institute for Cancer Research [32]. These cells were cultured in RPMI 1640 (Wako, Tokyo, Japan) with 10% fetal bovine serum (HyClone SH30910.03; GE Healthcare, Chicago, IL, USA) and penicillin-streptomycin (Sigma-Aldrich, St. Louis, MO, USA) at 37 °C in a 5% CO_2_ incubator. For *in vitro* EBV infection of MKN7 and GES1 cells, the Akata system was used as previously reported [10]. DNA was extracted using the QIAamp DNA Mini Kit (Qiagen, Hilden, Germany). RNA was extracted using the RNeasy Mini Kit (Qiagen) following the manufacturer's protocol, and treated with DNaseI (Qiagen).

### Plasmid construction

cDNA of *TET2* transcript variant 1 was inserted into the EcoRV site of the pcDNA5/TO vector. The CMV promoter region of pcDNA5/TO was changed to a CAG promoter and hygromycin resistance gene was changed to puromycin resistance gene. Beginning at 16 h before transfection, cells were cultured in RPMI 1640 medium with 10% fetal bovine serum without penicillin-streptomycin in 6-well dishes, and 2 μg of the TET2-expressing vector or mock vector were transfected using Lipofectamine 2000 (Invitrogen, Carlsbad, CA, USA). The transfectants were selected using 2 μg/mL puromycin (Sigma-Aldrich). As for EBV latent genes, MKN7 was trasnfected with pcDNA3 or pcDNA5/TO vector (Invitrogen) containing cDNA of *LMP2A*, *EBNA1*, *EBER1/2* and *BARF0* or mock vector, using lipofectamine 2000 (Invitrogen), and selected with 200 μg/mL geneticin or hygromycin B, as previously reported [7].

### Knockdown by shRNA

To knock down *TET2*, double-stranded oligonucleotide DNA encoding small hairpin RNA (shRNA) against *TET2* was cloned into the pLKO.1 vector between EcoRI and AgeI sites. Oligonucleotide sequences for shRNA against *TET2* (shTET2) and control non-target shRNA (shNON) are listed in Supplementary Table S2. Viral packaging for shRNA retrovirus vectors was performed using 293T cells and FuGENE 6 (Promega, Madison, WI, USA), and medium containing the virus was collected 48 h after transfection.

### Real-time RT-PCR

cDNA was prepared from 1 μg of total RNA using SuperScript III Reverse Transcriptase (ThermoFisher, Waltham, MA, USA). Real-time RT-PCR was performed using SYBR Green and CFX96 Touch Real-Time PCR (Bio-Rad Laboratories, Hercules, CA, USA). The quantity of mRNA for each gene in a sample was estimated by comparisons with standard samples that contained 10^1^ to 10^6^ gene copies. These levels were normalized to those of *GAPDH* and *PPIA*, as previously described [33]. The PCR primers and conditions are presented in Supplementary Table S3.

### Immunoblotting analysis

TET2 and the internal control α-Tubulin were detected by immunoblotting analysis using a rabbit anti-TET2 polyclonal antibody (1:1000, R1086-3, Abiocode, Agoura Hills, CA, USA) and a mouse anti-α-Tubulin monoclonal antibody (1:4000, sc-5286, Santa Cruz, Dallas, TX, USA). Protein-blotted membranes were incubated with antibodies using Can Get Signal Immunoreaction Enhancer Solution (Toyobo, Osaka, Japan) at 4 °C overnight for the primary antibodies, and at room temperature for 1 h for secondary antibodies, followed by visualization using the ECL prime system (GE Healthcare, Buckinghamshire, UK). The protein signals were detected using Luminescent Image Analyzer LAS-3000 (Fujifilm, Tokyo, Japan).

### Hydroxymethylated and methylated DNA immunoprecipitation sequencing (hMeDIP-seq/MeDIP-seq)

DNA regions with 5hmC and 5mC were analyzed by hMeDIP and MeDIP, respectively. Fragmentation of genomic DNA was performed using a Picoruptor (Diagenode, Seraing, Belgium) for 10 sets of 30 s on and 30 s off; 20 μg were prepared for hMeDIP and 4 μg were prepared for MeDIP. Fragmented DNA was separated into two tubes, and the tubes were incubated at 95 °C for 10 min followed by 10 min on ice. Immunoprecipitation (IP) Buffer was added to reach 500 μL; subsequently, 2 μL of anti-5hmC antibody (#39769; Active Motif, Carlsbad, CA, USA) was added for hMeDIP or 4 μL of anti-5mC antibody (#33D3; Diagenode) was added for MeDIP. The components of the buffer are described in Supplementary Table S4. The tubes were rotated at 4 °C overnight. Both 50 μL of 50% Protein A and G Sepharose (GE Healthcare) were added to each tube, followed by rotation at 4 °C for 2 h. The DNA-bead mixture was moved to columns (Corning, New York, NY, USA) and centrifuged at 1,000 × *g* for 1 min at 4 °C. After the flow-through was discarded, the beads were subjected to washing steps using 500 μL of IP Buffer twice, 500 μL of Wash Buffer 5 times, and 500 μL of TE Buffer twice. Beads were transferred to tubes with 400 μL of Elution Buffer and treated with 5 μL of Proteinase K (New England Biolabs, Ipswich, MA, USA) at 55 °C for 1 h. After phenol and chloroform DNA purification, DNA was eluted with 20 μL of distilled water. Enrichment of genomic regions in samples after hMeDIP and MeDIP was validated by real-time PCR using primers listed in Supplementary Table S5. The hMeDIP and MeDIP DNA were used to prepare library samples using the NEBNext ChIP-Seq Library Preparation Set for Illumina (New England Biolabs) following the manufacturer's protocol. Deep sequencing was performed on the Illumina HiSeq 1500 or NextSeq 500 system using the TruSeq Rapid SBS Kit (Illumina, San Diego, CA, USA) in 50-base single-end mode according to the manufacturer's protocol. The FASTQ reads were mapped to the hg19 reference sequence (UCSC) using BWA with default settings. The numbers of uniquely mapped reads for hMeDIP samples were 18,046,940 (Mock) and 16,171,239 (TET2 overexpression). For MeDIP samples, there were 16,322,113 (WT) and 17,482,843 (EBV) uniquely mapped reads. These hMeDIP-seq and MeDIP-seq data were submitted to the NCBI BioSample database (http://www.ncbi.nlm.nih.gov/biosample), and the accession numbers are GSM2253669 - GSM2253672. To count the number of reads that were mapped to within ±1 kb of transcription start sites (TSS), Count Reads version 0.2 was used with a window size of 300 bp, and the read count for each window was divided by the total read count and expressed as reads per million mapped sequence reads (RPM), as previously described [34]. Only high-CpG promoter genes (CpG score ≥0.48) [35] were analyzed.

### RNA-sequencing (RNA-seq) analysis

Libraries for RNA-seq were prepared using the TruSeq Stranded mRNA Sample Prep Kit (Illumina), following the manufacturer's protocol. Deep sequencing was performed on the Illumina HiSeq 1500 or NextSeq 500 platform using the TruSeq Rapid SBS Kit (Illumina) in 50-base single-end mode according to the manufacturer's protocol. The RNA-seq data were submitted to the NCBI BioSample database (http://www.ncbi.nlm.nih.gov/biosample), and the accession numbers are GSM2253673 - GSM2253676. TopHat was used to map FASTQ reads and Cufflinks was used for transcript assembly. Gene expression levels were expressed as fragments per kilobase of exon per million mapped sequence reads (FPKM). When expression alterations were analyzed, expression levels are presented as log2 FPKM values, excluding genes with log2 FPKM ≤0.

### Infinium assays

The Infinium HumanMethylation450 BeadChip (Illumina) contains approximately 485,000 individual CpG sites covering 99% of RefSeq genes with an average of 17 CpG sites per gene. In each CpG site, the ratio of the fluorescent signal, so-called β value, was measured by a methylated probe relative to the sum of both methylated and unmethylated probes [36]. The β values range from 0.00 to 1.00 and reflect the methylation level of each CpG site, from low to high. Bisulfite conversion was performed using the Zymo EZ DNA Methylation Kit (Zymo Research, Irvine, CA, USA) with 500 ng of genomic DNA for each sample. Whole genome amplification, labeling, hybridization, and scanning were performed according to the manufacturer's protocols. Genes were classified into the following four types based on methylation alterations within ±1 kb of a TSS. (i) “Unmethylated genes” contained >2 probes with β <0.2 in wild-type cells and no probes with β >0.4 after EBV infection. (ii) “Methylated genes” contained >2 probes with β <0.2 in wild-type cells and >2 probes with β from <0.2 to >0.4 after EBV infection. Among “Methylated genes,” (iii) “Methylation-sensitive genes” were those in which all probes showed β >0.2 after EBV infection, and (iv) “Methylation-resistant genes” were those in which >2 probes in a row showed β <0.2 even after EBV infection (Supplementary Figure S3).

In a bisulfite-based methylation assay, 5hmC and 5mC cannot be distinguished [37, 38]. Unmethylated cytosine changes to uracil, which is read as thymine by PCR, but neither 5hmC nor 5mC changes in response to bisulfite treatment, and both are read as cytosine. The quantity of 5hmC, however, is much smaller than that of 5mC. In this study, the average β value of hydroxymethylated genes with only a 5hmC peak and no 5mC peak was only 0.07, whereas that of methylated genes with only a 5mC peak and no 5hmC peak was 0.54. The results for representative genes are shown in Supplementary Figure S4. Because the β value for 5hmC was sufficiently small, an Infinium assay was performed for 5mC detection.

### Pyrosequencing analysis

Validation for methylated locus was carried out by pyrosequencing as described previously [7]. Primers for pyrosequencing were designed by Pyrosequencing Assay Design Software ver.2.0 (QIAGEN) to amplify bisulfite-treated DNA region containing several CpG sites. Primer sequences are listed in Supplementary Table S6.

### Analysis of miRNA

For the micro RNA (miRNA) expression analysis, Direct-zol RNA MiniPrep (Zymo Research) was used to extract total RNA, including miRNAs. The miRNA Microarray System with miRNA Complete Labeling and Hyb Kit Version 2.4 (Agilent Technologies, *Santa Clara, CA, USA*) was used with 100 ng of total RNA as an input, following the manufacturer's instructions. After washing, scanning was conducted using a DNA Microarray Scanner (Agilent Technologies) and the resulting image data were converted to numerical form using Feature Extraction ver. 10.7.1.1 (Agilent Technologies). The numerical data were normalized using GeneSpring GX 12.0 (Agilent Technologies). For validation, candidate miRNA in mature form was obtained (Bioneer, Daejeon, Republic of Korea) and transfected into MKN7 and GES1 with a final concentration of 20 nM using Lipofectamine 2000 (Invitrogen).

### Statistical analyses

Statistical analyses of gene expression and methylation levels based on β values were performed using Wilcoxon signed-rank test. Gene counts were compared using the χ^2^ test. R program (www.r-project.org/) was implemented in those testing. *P*<0.05 was considered to be statistically significant.

## SUPPLEMENTARY MATERIALS FIGURES AND TABLES


