# Supplementary material for: TET2 functions as a resistance factor against DNA methylation acquisition during Epstein-Barr virus infection
Source: Oncotarget. 2016 Nov 5;7(49):81512–26. doi: 10.18632/oncotarget.13130 (PMC5348409; doi:10.18632/oncotarget.13130)
Supplement: Supplementary file 1 [file oncotarget-07-81512-s001.pdf]

TET2 functions as a resistance factor against DNA methylation acquisition during Epstein-Barr virus infection

SUPPLEMENTARY FIGURES AND TABLES

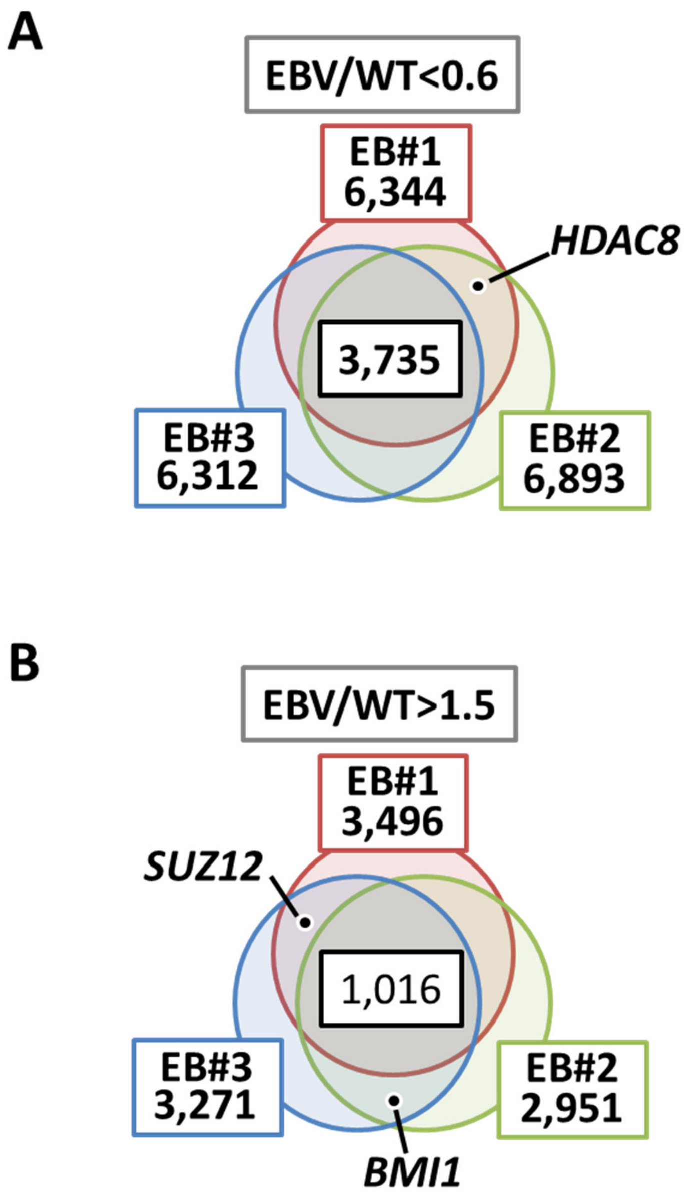

Supplementary Figure S1: The genes downregulated and upregulated by EBV infection. A. The epigenetic factors downregulated by EBV infection include *HDAC8*, which was downregulated in two of EBV-infected MKN7 clones. **B.** The epigenetic factors upregulated by EBV infection include *SUZ12* and *BMI1*, which were upregulated in two of EBV-infected MKN7 clones.

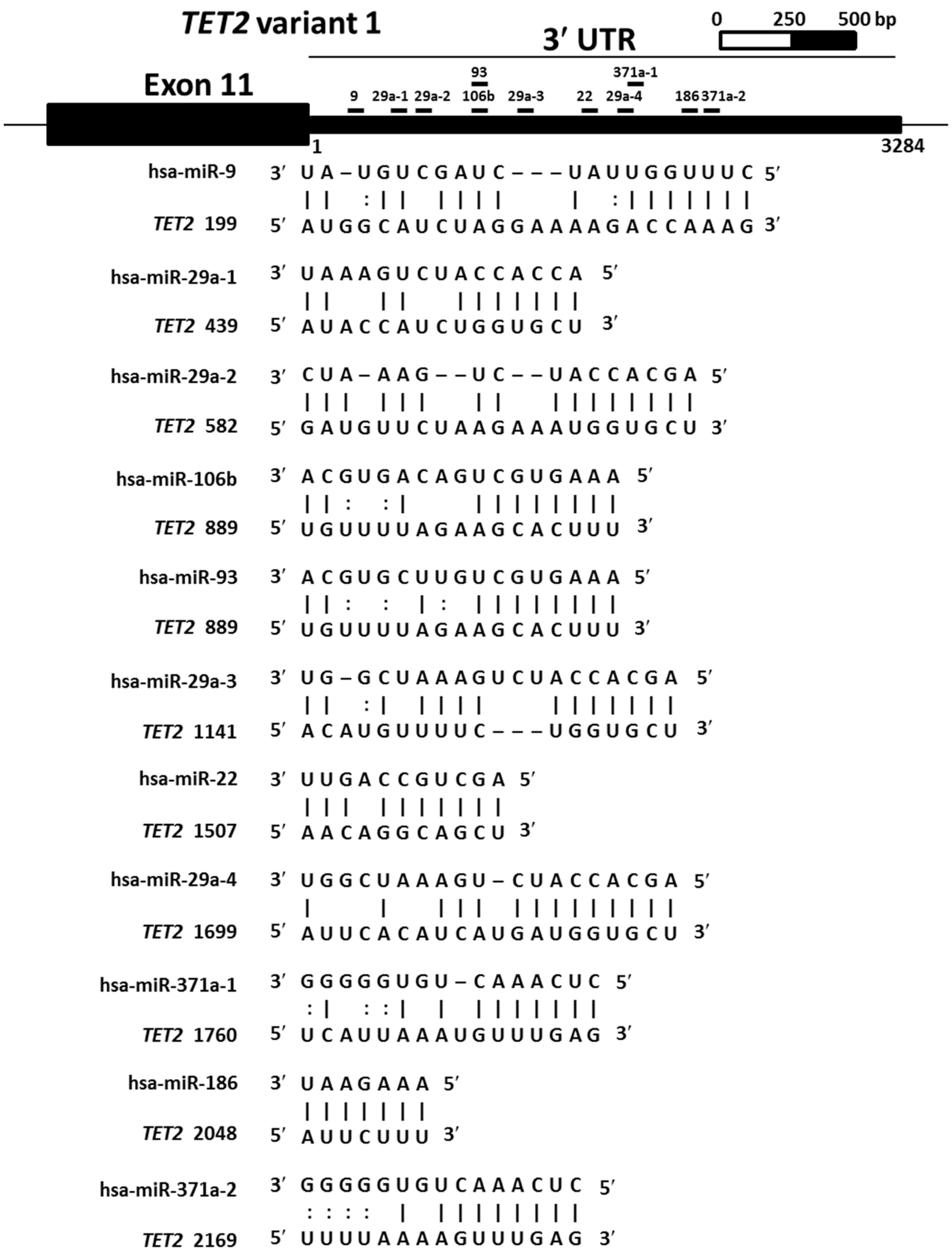

Supplementary Figure S2: The predicted target regions of 7 miRNAs targeting *TET2*. hsa-miR-29a has 4 sites and has-miR-371a has 2 sites to bind 3′ UTR of *TET2*.

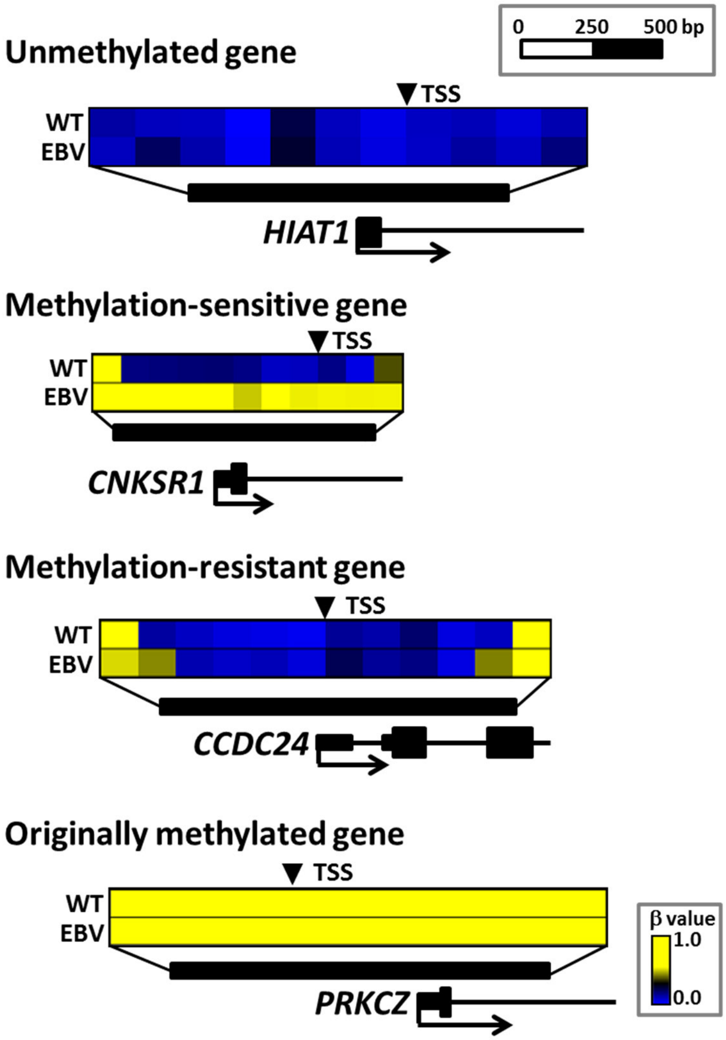

Supplementary Figure S3: Representation of an unmethylated gene (*HIAT1*), a methylation-sensitive gene (*CNKSR1*), a methylation-resistant gene (*CCDC24*), and an originally methylated gene (*PRKCZ*) during EBV infection. When *de novo* methylation is induced by EBV infection, there are two types of methylation acquisition around promoter regions. Methylation-sensitive genes undergo complete methylation around the promoter regions; methylation-resistant genes undergo methylation in regions surrounding their promoters, but maintain an unmethylated status in narrow regions around the TSS.

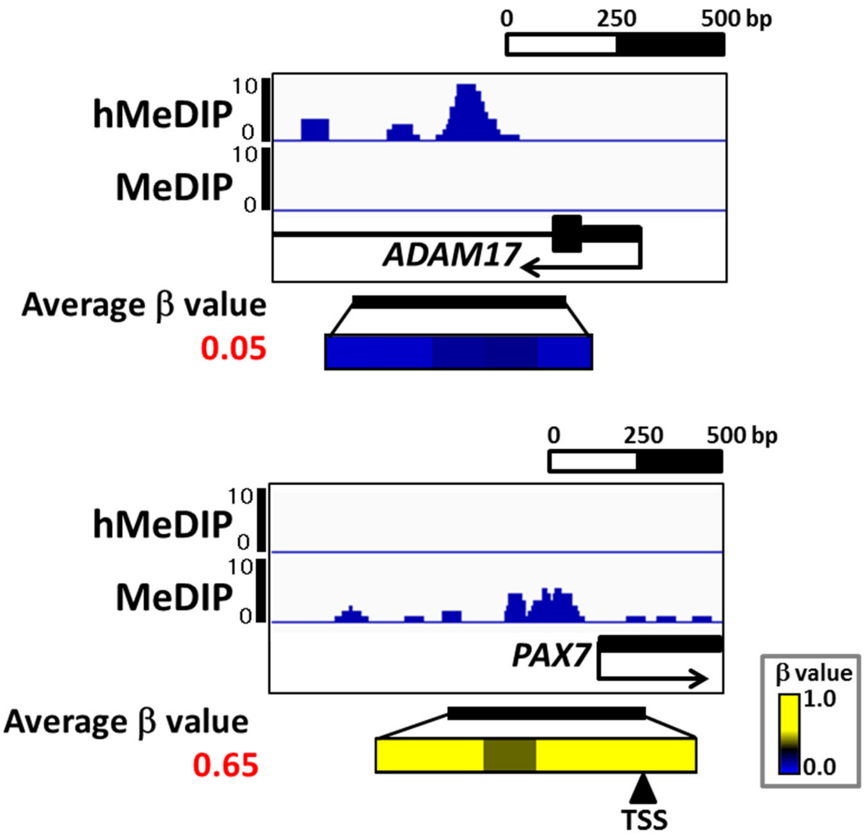

Supplementary Figure S4: Representative genes with an hmC peak, but no mC peak, e.g., *ADAM17*, and those with mC peak and no hmC peak, e.g., *PAX7*, are shown. The β values of the former genes were very low, e.g., 0.05 for *ADAM17*. The β values of the latter genes were markedly higher, e.g., 0.65 for *PAX7*.

Supplementary Table S1: EBV miRNA and the targeting human genes

See Supplementary File 1

Supplementary Table S2: shRNA target sequences in lentivirus

target gene	strand	sequence
TET2	top	CCGGCAGATGCACAGGCCAATTAAGCTCGAGCTTAATTGGCCTGTGCATCTGTTTTTG
	bottom	AATTCAAAAACAGATGCACAGGCCAATTAAGCTCGAGCTTAATTGGCCTGTGCATCTG
Non-target	top	CCGGCAACAAGATGAAGAGCACCAACTCGAGTTGGTGCTCTTCATCTTGTTGTTTTTG
	bottom	AATTCAAAAACAACAAGATGAAGAGCACCAACTCGAGTTGGTGCTCTTCATCTTGTTG

Oligonucleotide sequences of shRNA against *TET2* (shTET2) and control non-target shRNA (shNON), inserted into pLKO.1 vector between EcoRI and AgeI sites, are shown, and 21-mer target sequences are underlined.

Supplementary Table S3: Primer sequences for real-time RT-PCR

Gene	Primer types	Primer Sequence	Anneal (°C)	Product (bp)
TET1	Forward	CTTACAGAGTTTGGCTACACGAT	60	93
	Reverse	CGGGCAACATTTTCATATTCCAC		
TET2	Forward	ATTGAAACAAGACCAAAAGGCTA	60	109
	Reverse	CTTATCACTCAAATCGGAGACA		
TET3	Forward	CCACGGCTTCCAGACAGACCAC	60	91
	Reverse	CAGGGGCAGCTTTCTCCGTTCCCA		
GAPDH	Forward	CCAGGTGGTCTCCTCTGACTTC	60	108
	Reverse	TCATACCAGGAAATGAGCTTGACA		
PPIA	Forward	ACAGTGCTTGCTGGCAGTTAGA	60	130
	Reverse	CAAATCCGCCACCTCTAGGATAG		

Supplementary Table S4: Buffers for DIP experiment

IP Buffer	20 mM tris-HCl pH8.0
	2 mM EDTA
	150 mM NaCl
	1.0% Triton-X100
Wash Buffer	20 mM Tris-HCl pH8.0
	2 mM EDTA
	300 mM NaCl
	0.1% SDS
	1.0% Triton-X100
Elution Buffer	25 mM Tris-HCl pH8.0
	10 mM EDTA
	0.5% SDS
	100 mM NaCL

Supplementary Table S5: Primers for hMeDIP/MeDIP-PCR

Gene	Primer types	Primer Sequence	Anneal (°C)	Product (bp)	hMe/Me
Positive control regions					
NEDD9	Forward	GCTTCATGCCTGCTACATATACTCC	60	106	hMe, Me
	Reverse	TGTGGTCTGTTTGTGTGTAAGAGG			
HMGA2	Forward	CATGCAGAGCAGCGATTTAAA	60	99	hMe
	Reverse	GCCTTTCTTCTTGCTTTAGGACAA			
ACTB	Forward	GAGGGAAATGAGGGCAGGACTT	60	127	hMe
	Reverse	GCTGCCCTGAGGCACTCTTC			
AJAP1	Forward	CGAGCCAGGTCTGAGGC	60	77	Me
	Reverse	GGTCGCTCACCTGAGTCCTA			
Negative control region					
HBB	Forward	GGGCTGAGGGTTTGAAGTCC	60	89	hMe, Me
	Reverse	CCACAGGGTGAGGTCTAAGTG			
Validation region					
FRG1B	Forward	CTTCCTCCATCTCTATCGGGCAA	60	73	hMe
	Reverse	CTAATCCTGGAGGCGGCATCTGA			

Supplementary Table S6: Primers for pyrosequencing

Gene	strand	Primer types	Primer Sequence	Anneal (°C)	Product (bp)
FRG1B	bottom	Forward	GAGTGTTGTGGGAATGTGG	56	101
		Reverse*	TCCTCAAATACCCCCTCCAAAATTAAACT		
		Sequencing	GGTGGAGAGTTATGGT		
HTRA1	bottom	Forward*	GGAGAGTGTAGGAGGGTTT	58	134
		Reverse	CCATCCCACCAACCCCCATC		
		Sequencing	CACCCCTACCAAACCAATAAACT		

* Primers with 5′-biotin tag.
